# Inbreeding estimates in human populations: Applying new approaches to an admixed Brazilian isolate

**DOI:** 10.1371/journal.pone.0196360

**Published:** 2018-04-24

**Authors:** Renan B. Lemes, Kelly Nunes, Juliana E. P. Carnavalli, Lilian Kimura, Regina C. Mingroni-Netto, Diogo Meyer, Paulo A. Otto

**Affiliations:** Department of Genetics and Evolutionary Biology, Instituto de Biociências, Universidade de São Paulo, São Paulo, São Paulo, Brazil; Universitat Pompeu Fabra, SPAIN

## Abstract

The analysis of genomic data (~400,000 autosomal SNPs) enabled the reliable estimation of inbreeding levels in a sample of 541 individuals sampled from a highly admixed Brazilian population isolate (an African-derived *quilombo* in the State of São Paulo). To achieve this, different methods were applied to the joint information of two sets of markers (one complete and another excluding loci in patent linkage disequilibrium). This strategy allowed the detection and exclusion of markers that biased the estimation of the average population inbreeding coefficient (Wright’s fixation index *F*_*IS*_), which value was eventually estimated as around 1% using any of the methods we applied. *Quilombo* demographic inferences were made by analyzing the structure of runs of homozygosity (ROH), which were adapted to cope with a highly admixed population with a complex foundation history. Our results suggest that the amount of ROH <2Mb of admixed populations should be somehow proportional to the genetic contribution from each parental population.

## Introduction

Measures of population inbreeding levels have been traditionally obtained from the direct genotyping of population samples followed by the estimation of heterozygous frequency deviations from the proportions expected under random-mating assumptions (HW expectations) or from the analysis of sets of individual or grouped genealogies (v.g., Lemes *et al*. [1]). The inbreeding coefficients *F* estimated from the two methods are however quantitatively and qualitatively different, since the first one (Wright's fixation index *F*_*IS*_) estimates specifically systematic inbreeding or consanguinity, while the second (Wright's fixation index *F*_*IT*_) measures the amount of total inbreeding, including the fraction due to small population effective number *Ne*. In humans, good examples of the usefulness of deep genealogies to measure inbreeding coefficients are the study of the isolated African-derived community of Valongo in Brazil [1,2] and the research on royal inbreeding [3,4]. Ceballos and Alvarez [4] study showed that it is possible to capture 95% of the actual inbreeding coefficient with a pedigree of at least 10 generations depth. On practical grounds, however, only in rare instances it is possible to include precise relationship information on more than three or four generations.

The situation has changed dramatically with the recent use of large datasets of genomic autosomal single nucleotide polymorphisms (SNPs), allowing the identification of long tracts of consecutive homozygosity (runs of homozygosity or ROH) in human population samples. Studies using this approach have revealed high levels of autozygosity even in cosmopolitan non-inbred populations, showing that there exists, as expected by the out-of-Africa model of human origins, an increment of inbreeding levels and a significant reduction of genetic diversity which is proportional to the geographic distance from Africa [5–7]. An important mechanism contributing to a large portion of genomic homozygosity levels, composed mainly by short and intermediate-sized ROH, is background relatedness, which results from the combined effects of demographic and evolutionary events, such as remote inbreeding, geographic isolation, small population size with bottleneck and founder effects, and long-lasting and stable systems of endogamous marriages due to the persistence of cultural traditions [5,7–10].

Population isolates are powerful tools for medical and evolutionary studies, since many of them have well documented pedigrees, high prevalence of individuals affected by rare genetic conditions, high degree of inbreeding due to cultural practices or limited population size, and a demographic history of foundation consisting of bottlenecks followed by founder effects [11]. Even in the case of population isolates without well documented pedigrees and a paucity of historical records, reliable genetic information can be obtained from the analysis of large SNP datasets. Several studies of inbreeding and demographic history have been successfully performed around the world in isolated populations with variable amounts of genealogical documentation and historical records of population-based evolutionary phenomena [8,12–16].

The admixture of populations with different genetic backgrounds can create high levels of linkage disequilibrium (LD), which, in addition to taking many generations to disappear, will interfere with the distribution of ROH. Studies on LD have shown that haplotype sizes rarely surpass 100kb in humans and that total individual ROH lengths measured, with or without LD pruning, are the same when considering ROH longer than 1.5Mb [8,17–18].

Inbreeding levels are thus informative about a population’s history of admixture events, demography, and can also be related to its genetic load and to the prevalence of genetic diseases. Consequently, the reliable estimation of inbreeding levels is of central importance to human population genetics.

By means of the analysis of a dataset of genomic autosomal single nucleotide polymorphisms (SNP), we make inferences on inbreeding levels and demographic history of a Brazilian isolate with about 40% African, 39% European and 21% Native American contribution [19]. This study presents: (1) an alternative way to estimate the population inbreeding coefficient (Wright’s fixation index *F*_*IS*_), based solely on the analysis of a high-density SNP array, in order to compare its statistical parameters with the individual estimates obtained from software PLINK v1.9 [20]; (2) the application of a sliding window approach to identify ROH in a population that underwent a complex demographic history with tri-hybrid ancestral contribution; (3) a comparison between individual estimates of the inbreeding coefficient obtained from ROH data (equivalent to *F*_*IT*_) and from software PLINK v1.9 (equivalent to *F*_*IS*_).

## Subjects and methods

### The Brazilian *Quilombo* (QUI) admixed population

**Fig 1:**
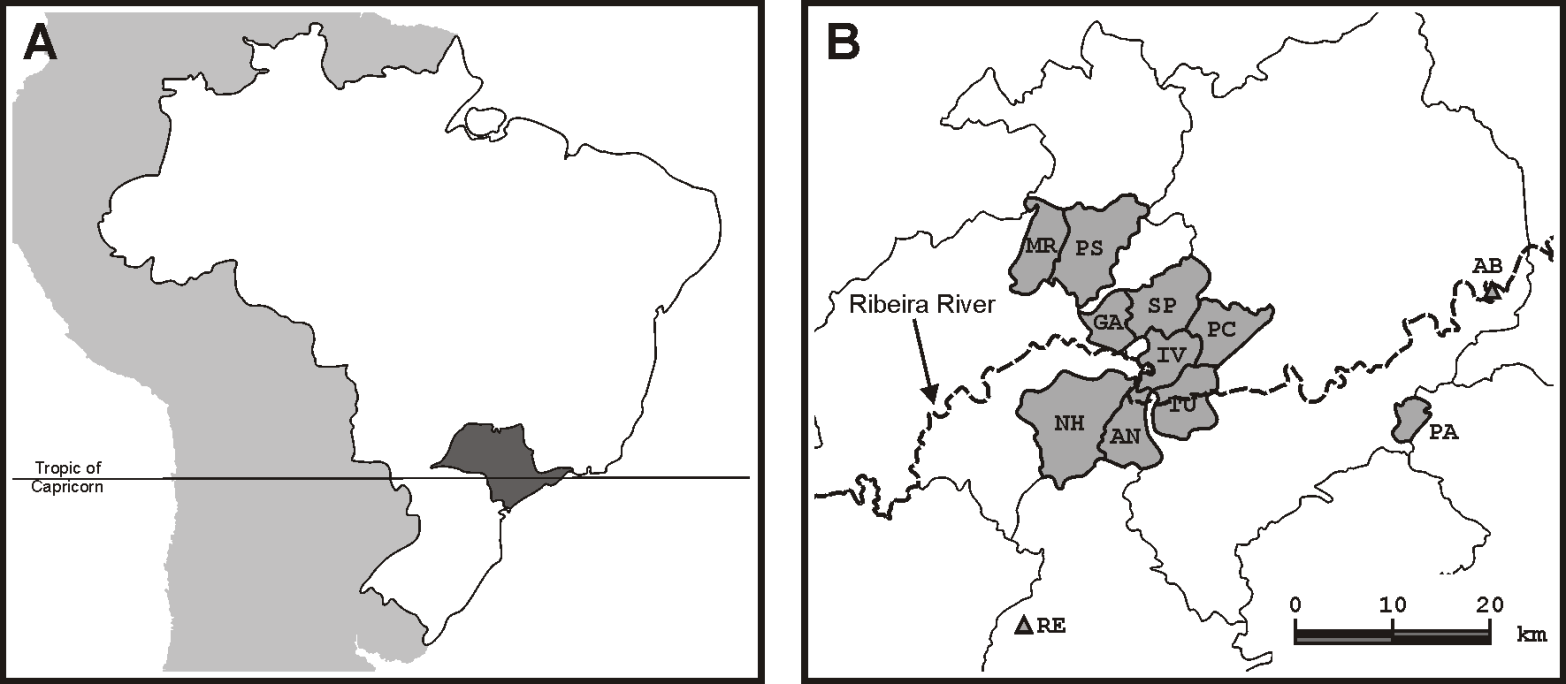
Location of *quilombo* communities. (A) State of São Paulo (gray) within Brazilian territory in South America. (B) Location of quilombo communities. AB, Abobral; AN, André Lopes; GA, Galvão; IV, Ivaporanduva; MR, Maria Rosa; NH, Nhunguara; PA, Poça; PC, Pedro Cubas; PS, Pilões; RE, Reginaldo; SP, São Pedro; TU, Sapatu. (Adapted from Lemes *et al*. [1]).

The present study was performed in an admixed Brazilian isolate located in the Ribeira River Valley, in the southern part of the State of São Paulo, Brazil (Fig 1). This isolate, known in Brazil as a *quilombo*, was founded around 1890 by runaway, abandoned and freed slaves (some of them being the admixed offspring of white farm owners and African female slaves) and a few pure or mixed native Americans, who created small rural settlements in isolated areas inside the Atlantic rainforest for several generations (other details of interest on the *quilombo* population structure and demography are described elsewhere [1,19,21]). The isolate aggregates twelve communities that were treated as a single one, since the degree of differentiation among its communities is very low, with *F*_*ST*_ indices generally smaller than 0.05 [1].

Some fifty years ago a road was built near the communities and a significant degree of migration between neighboring populations began to take place. Because of this recent history of admixture, some people argue that the quilombo reported here does not represent a true isolate anymore. In order to warrant or preserve the isolate condition with which we classify this population aggregate, all individuals selected for this study, aged between 17–65 years, have at least two generations of local quilombo ancestors.

DNA samples were extracted from peripheral blood and genotyped with the SNP array Axiom Genome-Wide Human Origins (~600,000 SNPs) according to the manufacturer's standards (Affymetrix/Thermo-Fisher Scientific). We analyzed DNA samples from 541 individuals (S1 Table) from the Ribeira River Valley, 365 of them having already been genotyped in a previous study [22] and the remaining 176 samples of this study. The research was approved by the Ethics Committee, Instituto de Ciências Biomédicas, Universidade de São Paulo (111/CEP, Feb. 14^th^ 2001), and an informed consent was obtained from all its participants or their legal guardians.

### HGDP samples

Data of 934 humans were selected from dataset 11 of the Human Genome Diversity Project (HGDP), which includes individuals from Africa (105), Europe (151), Middle East (160), Central South Asia (197), East Asia (231), Oceania (28), and America (61), many of them from population isolates. This sample was also genotyped for the same set of ~600,000 SNPs described in the section above and is available at ftp://ftp.cephb.fr/hgdp_supp10/Harvard_HGDP-CEPH/.

### Data preparation

In order to minimize the effects of genotyping error, we carried out in the QUI dataset a process of data cleaning which excluded: (1) all markers with low quality scores, using the software Genotype Console Software v.4.2 according to the manufacturer's standards parameters (Genotype Console Workflow–Affymetrix/Thermo Fisher Scientific); (2) all markers with significant differences in missing data proportions between groups (defined by sex, experimental batch, and subpopulation status) using the R package GWASTools v.3.5 [23]; (3) all genotyped loci with more than 10% of missing values; (4) all data from loci with extreme deviations from Hardy-Weinberg proportions (P ≤ 10^−8^), using the asymptotic exact test [24] by means of the software PLINK v1.9 [20]. We also excluded (1) all data from autosomal triallelic markers, mitochondria and X and Y chromosomes (X markers were excluded because after data cleaning and merging their number was critically reduced); (2) all markers with minor allele frequency (MAF) of 0, i.e., all alleles that were fixed; and (3) all data corresponding to markers within the 2Mb of the extremities of all chromosome arms, for which genotyping is less reliable. The final QUI set consisted of data from 477,426 autosomal SNPs.

We also excluded loci data of each HGDP population having more than 10% of missing values, extreme deviations from Hardy-Weinberg proportions (P ≤ 10^−8^), or within 2Mb from the extremities of all chromosome arms. The final HGDP set was merged with QUI, consisting of data from 388,702 autosomal SNPs.

### Estimation of the inbreeding coefficient (Wright's fixation index *F*_*IS*_)

With the aim of comparing their statistical parameters (mean, median, variance, and 95% approximate and 'exact' confidence intervals), Wright's fixation index *F*_*IS*_ was estimated using two different methods (detailed in the paragraphs below). The first method obtains the population inbreeding coefficient averaging the fixation indices estimated from each locus of all sampled individuals; in the second one the population inbreeding coefficient is obtained by averaging the fixation indices indirectly obtained from all sampled loci of each individual. As one can guess, the two methods should be *a priori* grossly equivalent, but (as stressed before) we are interested only in comparing their corresponding parameters with the obvious aim to verify whether one out of the two might be occasionally more appropriate, adequate or convenient to use. As far as we can tell, this has not been performed in the literature before.

To obtain the average estimates (across all loci of all individuals from QUI sample) of Wright's fixation index *F*_*IS*_ we used the information from (1) all 477,426 SNPs (complete dataset) and (2) 11,321 SNPs with no LD (no-LD dataset), obtained using the software PLINK v1.9 [20], considering a threshold of r^2^ = 0.0071, which corresponds to a critical 5% chi-square value of χ^2^ = 3.841, pairwise estimated in sliding windows of 50 SNPs incremented in steps of 5.

#### First estimate of Wright's *F*_*IS*_ coefficient

Using the first method (described above), the inbreeding coefficient *F*_*IS*_ = *f*_*k*_ was obtained for each biallelic locus by means of the classical and basic formula

$$f_{k}=1-\frac{P_{k}\left(Aa\right)}{2p_{k}q_{k}},$$ (1)

where *P*_*k*_*(Aa)* and *2p*_*k*_*q*_*k*_ are respectively the observed and HW expected frequencies of heterozygous genotypes at the k-th locus. The mean population inbreeding coefficient ($\bar{f}$) over all loci was obtained weighing the per locus *f*_*k*_ estimates by the reciprocals of their corresponding variances:

$$\bar{f}=\sum x_{k}.f_{k},$$ (2)

with

$$x_{k}={var}^{‑1}\left(f_{k}\right)/\sum_{j=1}^{n}{var}^{‑1}\left(f_{j}\right),$$ (3)

where *n* is the number of loci and *var(f*_*k*_*)* is the estimate of the variance of *f*_*k*_, obtained for each biallelic locus by the formula [25–27]:

$$var(f_{k})=\frac{\left(1-f_{k}\right)\left[2p_{k}q_{k}+2f_{k}\left(1-3p_{k}q_{k}\right)-f_{k}{}^{2}\left(1-4p_{k}q_{k}\right)\right]}{2Np_{k}q_{k}},$$ (4)

where *N* is the sample size, and *p*_*k*_ and *q*_*k*_ are the frequencies of the alleles segregating at the k-th bialellic SNP locus.

In the long run, one expects that the estimates of *f*_*k*_ thus obtained should be normally distributed around the average value of $\bar{f}$, with the limits of the usual 95% confidence interval being given approximately by $\bar{f}\pm 1.96\sqrt{var\left(f\right)}$, where *var(f)* is given by

$$var\left(f\right)={\sum x_{k}f}_{k}^{2}-{\bar{f}}^{2},$$ (5)

with *x*_*k*_ as defined in formula (3) [1].

We also ranked the values of *f*_*k*_ in order to obtain the median and its observed (‘exact’) 95% confidence interval corresponding to the set of all values between the limits of the 2.5^th^ and 97.5^th^ percentiles.

#### Second estimate of Wright's *F*_*IS*_ coefficient

The estimate of the inbreeding coefficient *F*_*IS*_ for each individual of the sample, referred here as *f'*_*i*_, was obtained by means of the function —*het* of the software PLINK v1.9 using the expression:

$$f_{i}^{\prime }=\frac{\left(O_{i}{‑E}_{i}\right)}{\left(L_{i}{‑E}_{i}\right)},$$ (6)

where *O*_*i*_ and *E*_*i*_ are the observed and expected numbers of homozygous genotypes considering all *L*_*i*_ genotyped autosomal SNPs of individual *i* [20]. The mean value ($\bar{f}\prime$) was obtained by averaging all *f'*_*i*_ estimates; the corresponding 95% confidence interval of the whole distribution were obtained either using a normal approximation as before, or by ranking all the individual values.

### Identification of runs of homozygosity (ROH)

The identification of ROH was performed in the merged data (QUI + HGDP) by means of the software PLINK v1.9 [20], a method that has been successfully applied in many studies, enabling meaningful comparisons between different populations, cohorts, and array chips [17,18]. The algorithm is known to be also able to provide reliable ROH calls even when using data from next generation sequencing [28,29].

We considered here the same criteria described by McQuillan *et al*. [8] and Kirin *et al*. [5]: a sliding window with 50 SNPs; a maximum of one heterozygous genotype and five missing calls allowed per window; a proportion of windows that overlap to form an homozygous segment of 5%; a density of at least one SNP per 50kb; and a maximum gap between consecutive SNPs of 100kb. All the analysis was performed considering three different sets of ROH, identified considering minimum lengths of 500kb, 1.5Mb, and 5Mb.

### Estimation of inbreeding coefficient from ROH

Individual and population inbreeding coefficients were also estimated using ROH data. The *F*_*ROH*_, defined as the genomic autosomal proportion of ROH of an individual, was estimated by the expression [8]:

$$F_{ROH}=\frac{\sum L_{ROH}}{L_{auto}},$$ (7)

where *ΣL*_*ROH*_ corresponds to the length of ROH and *L*_*auto*_ corresponds to the total genomic region covered by the SNP array. Averaging the values of all individual F_ROH_s we obtain a parameter that is equivalent to Wright's fixation index *F*_*IT*_.

## Results

### Estimation of the average inbreeding coefficients *f* and *f'*

For the estimation of the first coefficient (*f*) we performed the analysis of complete and no-LD datasets using two approaches: (1) obtaining the $\bar{f}$ estimates for subsets of markers in different MAF (minor allele frequency) bins; and (2) observing the behavior of per locus estimates of *f*_*k*_.

**Fig 2:**
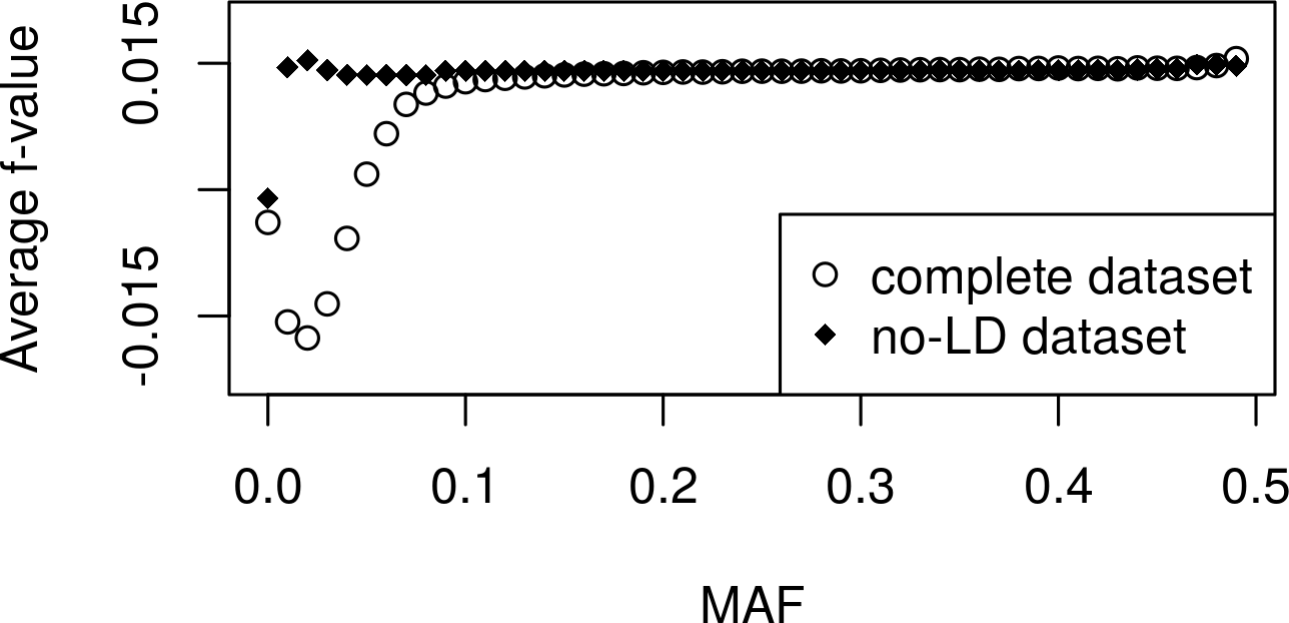
Average *f*-values. Average *f*-values corresponding to subsets of markers with MAF value equal or above the value shown in the abscissa axis.

Average values were estimated for subsets of markers according to thresholds of MAF ≥ {0, 0.01, …, 0.49}, showing a marked shift to negative values for markers with MAF < 0.1, and tendency to reach a plateau for MAF values close to 0.2 and higher (Fig 2).

**Fig 3:**
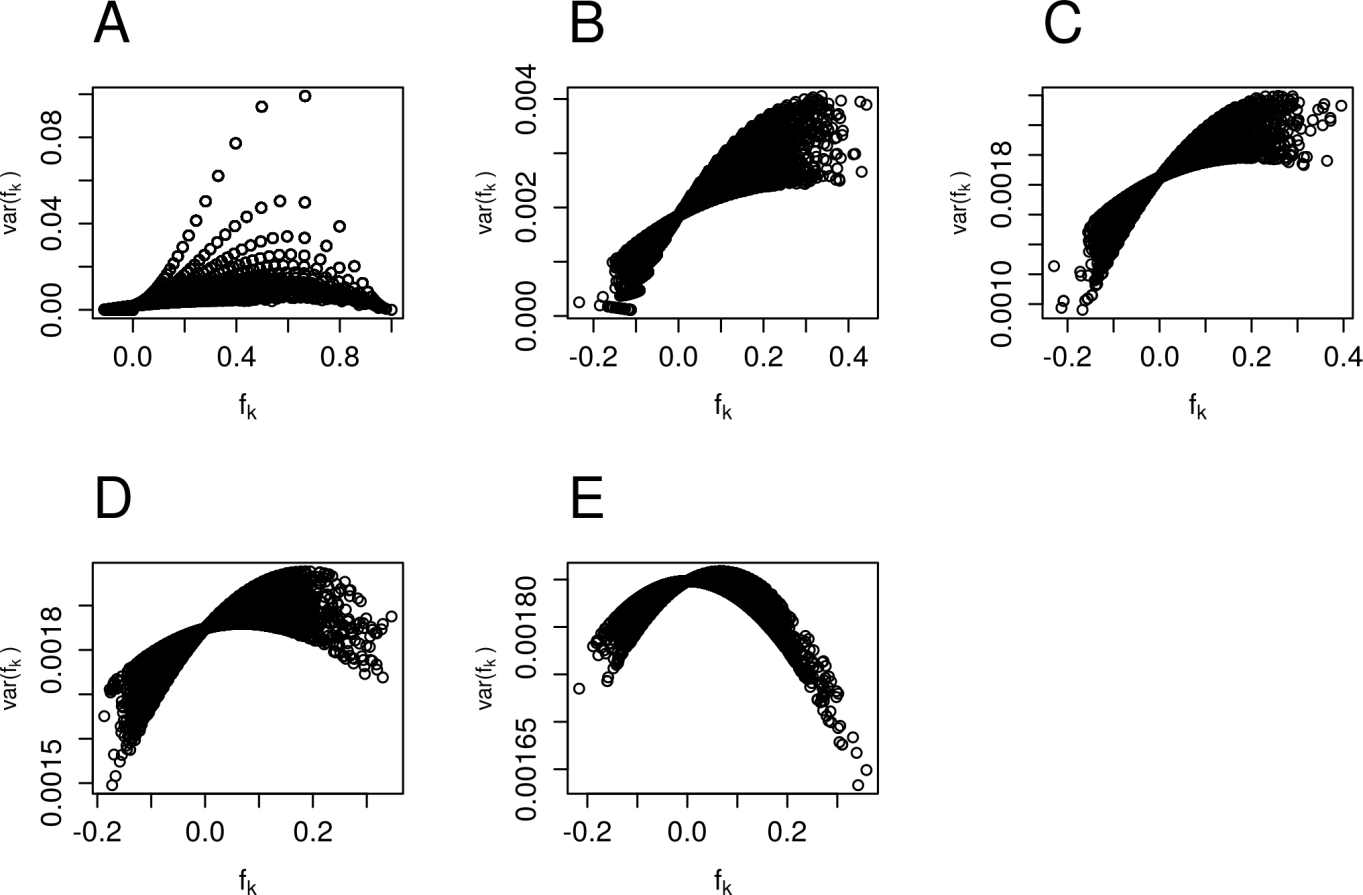
Variance of inbreeding coefficient per locus. Scatter plot of per locus *var(f*_*k*_*)* estimates and their corresponding *f*_*k*_ values according to MAF intervals for the complete dataset. (A) 0-0.1; (B) 0.1-0.2; (C) 0.2-0.3; (D) 0.3-0.4; (E) 0.4-0.5.

Considering now the behavior of individual *f*_*k*_ estimates across all loci of both datasets (S1 Fig), we notice that in spite of a huge amount of estimates obtained from markers with MAF < 0.1 holding positive values, almost half of *f*_*k*_ estimates have near zero and negative values. While these positive *f*_*k*_ values are associated with larger *var(f*_*k*_*)*, the negative ones are associated to much smaller values of *var(f*_*k*_*)*, some of them also very near zero (Fig 3). It makes clear the existence of negative values of *f*_*k*_ with very small variance values responsible for creating biased average $\bar{f}‑\mathrm{value}$, since the average value of *f*_*k*_ is calculated after $\bar{f}=\sum x_{k}.f_{k}$, where $x_{k}=\frac{{var}^{‑1}\left(f_{k}\right)}{\sum_{j=1}^{n}{var}^{‑1}\left(f_{j}\right)}$ (formulae 2 and 3).

We also observed that the smallest values of MAF are associated with highly heterogeneous *var(f*_*k*_*)* values (S2 Fig). The *f*_*k*_ values associated with lowest *var(f*_*k*_*)* estimates are strongly influencing the estimation of $\bar{f}$, probably being responsible for preventing the average $\bar{f}‑\mathrm{value}$ to reach the plateau shown for higher MAF values in Fig 2.

Therefore, an increase in the proportion of heterozygotes and in negative *f*_*k*_ values is associated with estimates of *var(f*_*k*_*)* close to zero, whose reciprocal values are incredibly large, thus creating a significant bias in the estimation of the population average $\bar{f}‑\mathrm{value}$, even when occurring in relation to a very few number of loci.

Taking into account the facts above and the results shown in Fig 2, in order to avoid the use of markers associated with obvious biases in the estimation of the average inbreeding coefficient $\bar{f}$, we considered in our final analysis, presented in the paragraph below, only loci with MAF ≥ 0.2.

In spite of having their original datasets dramatically reduced in size (the complete one from 477,426 to 232,240 SNPs and the no-LD one from 11,321 to 9,026 SNPs), the *f*_*k*_-values virtually retained their original properties of being symmetrically and normally distributed around their mean and median estimates. Taking into account that both sets were cleaned from most of their biases and errors, the parameters extracted from them (shown in Table 1 below) surely constitute much more reliable estimates.

**Table 1:** Average inbreeding coefficients (*f* and *f’*) estimates.

	*Dataset*	*Mean*	*var(f)*	*Theoretical 95% c*.*i*.	*Median*	*Observed 95% c*.*i*.
*f*	*Complete*	0.0139	0.0026	(-0.0869, 0.1147)	0.0143	(-0.0806, 0.1191)
	*no-LD*	0.0141	0.0026	(-0.0853, 0.1131)	0.0128	(-0.0811, 0.1158)
*f’*	*Complete*	0.0114	0.0011	(-0.0531, 0.0758)	0.0058	(-0.0329, 0.0971)
	*no-LD*	0.0112	0.0010	(-0.0514, 0.0738)	0.0056	(-0.0370, 0.0986)

Average values of *f* and *f’*, medians, corresponding variances and 95% confidence intervals obtained for the two cleaned datasets. The (approximate) theoretical 95% confidence intervals were constructed under Gaussian assumptions and the (empirical) observed ones, as well as their medians, were obtained by ranking all individual *f*_*k*_-values.

The average population ${\bar{f}}^{\prime }$ value (obtained averaging the estimates of *f’*_*i*_ from QUI sample using the no-LD SNP dataset) was 0.0112; the median, obtained from the whole *f’*_*i*_ distribution, was 0.0056, with corresponding 95% confidence interval limits of -0.0370 and 0.0986 (Table 1). The limits of the 95% c.i. using a normal approximation were respectively -0.0514 and 0.0738. Interestingly, these estimates are not very different from those obtained using the traditional methods mentioned above.

The two methods, as previously guessed, are equivalent, since the estimates of the inbreeding coefficient obtained from them are of the same order of magnitude. The first method (that averages the fixation indices estimated from each locus of all sampled individuals), however, in order that non-biased estimates of *f* be avoided, should be applied to a dataset excluding all markers with a MAF < 0.2, at least for our population. Also, comparing, the corresponding statistical parameters of both *f* and *f'*, we notice that the variance of *f'* is significantly lower than that of *f*, at least when dealing with sample the sizes of ours. In any case, it seems that the second method (that averages the fixation indices indirectly obtained from all sampled loci of each individual) seems to be, on practical grounds, more convenient to use than the first one.

### Inbreeding and demographic inferences from ROH

The inbreeding coefficients *F*_*ROH*_ of all individuals of the 52 populations (51 from HGDP + QUI) were assessed separately and grouped in continental regions and considering ROH above three thresholds (0.5, 1.5 and 5Mb). In all cases, as expected by the out-of-Africa model of human origins, African and Native American average *F*_*ROH*_ estimates (mean and median) were the lowest and the largest values, respectively (Table 2), given that African are the most diverse human populations, while Native American are the least one. For ROH above 0.5Mb, Quilombo have the second lowest *F*_*ROH 0*.*5*_ estimate, suggesting a high amount of genomic variability, probably influenced by the process of admixture. Considering the ROH cut-off of 1.5MB, the QUI average *F*_*ROH 1*.*5*_ estimate is higher than the obtained for Africa and Europe and close to the Asian one, a pattern similar to that observed for ROH above 5Mb (*F*_*ROH 5*_). As shown by McQuillan *et al*. [8], *F*_*ROH 1*.*5*_ correlates the best with estimates obtained from pedigree analysis. Our results show that quilombo populations have, on average, an estimate of *F*_*ROH 1*.*5*_ a bit higher than the one corresponding to the progeny of third cousins.

**Table 2:** Estimates of inbreeding coefficient from ROH.

	*Region*	*Mean*	*Median*	*var(F*_*ROH*_*)*	*Observed (‘exact’) 95% c*.*i*.
*0*.*5Mb*	*Quilombo*	0.0480	0.0418	0.0006	(0.0212, 0.1153)
	*Africa*	0.0308	0.0272	0.0001	(0.0192, 0.0500)
	*Europe*	0.0832	0.0808	0.0001	(0.0682, 0.1087)
	*Middle East*	0.0877	0.0801	0.0009	(0.0461, 0.1462)
	*Asia*	0.0958	0.0962	0.0006	(0.0581, 0.1619)
	*Oceania*	0.1752	0.1767	0.0003	(0.1307, 0.1982)
	*America*	0.2061	0.1907	0.0033	(0.1128, 0.3021)
*1*.*5Mb*	*Quilombo*	0.0193	0.0111	0.0005	(0.0007, 0.0882)
	*Africa*	0.0095	0.0074	0.0001	(0.0007, 0.0285)
	*Europe*	0.0112	0.0081	0.0001	(0.0027, 0.0399)
	*Middle East*	0.0297	0.0208	0.0007	(0.0025, 0.0859)
	*Asia*	0.0212	0.0109	0.0006	(0.0030, 0.0999)
	*Oceania*	0.0379	0.0376	0.0001	(0.0184, 0.0559)
	*America*	0.0705	0.0537	0.0026	(0.0121, 0.1737)
*5Mb*	*Quilombo*	0.0182	0.0111	0.0003	(0.0022, 0.0745)
	*Africa*	0.0068	0.0047	0.0001	(0.0021, 0.0240)
	*Europe*	0.0098	0.0051	0.0001	(0.0020, 0.0318)
	*Middle East*	0.0236	0.0179	0.0004	(0.0020, 0.0695)
	*Asia*	0.0214	0.0095	0.0006	(0.0021, 0.0889)
	*Oceania*	0.0094	0.0071	0.0001	(0.0020, 0.0168)
	*America*	0.0481	0.0435	0.0011	(0.0023, 0.1018)

Mean, median and corresponding observed 95% confidence intervals of individual inbreeding coefficients *F*_*ROH*_ per continent, considering ROH above a minimum threshold.

The *F*_*ROH 1*.*5*_ estimates were also obtained considering each population separately (S2 Table). The QUI average *F*_*ROH*_ values are much lower than those obtained for other isolates like Native Americans Karitiana (~0.10) and Surui (~0.15), but very similar to the estimates from African isolates like Biaka (~0.016) and Mbuti pygmies (~0.014), and San (~0.018), that showed to be at least twice the value observed for Bantu (~0.007), Mandenka (~0.005), and Yoruba (~0.004).

**Fig 4:**
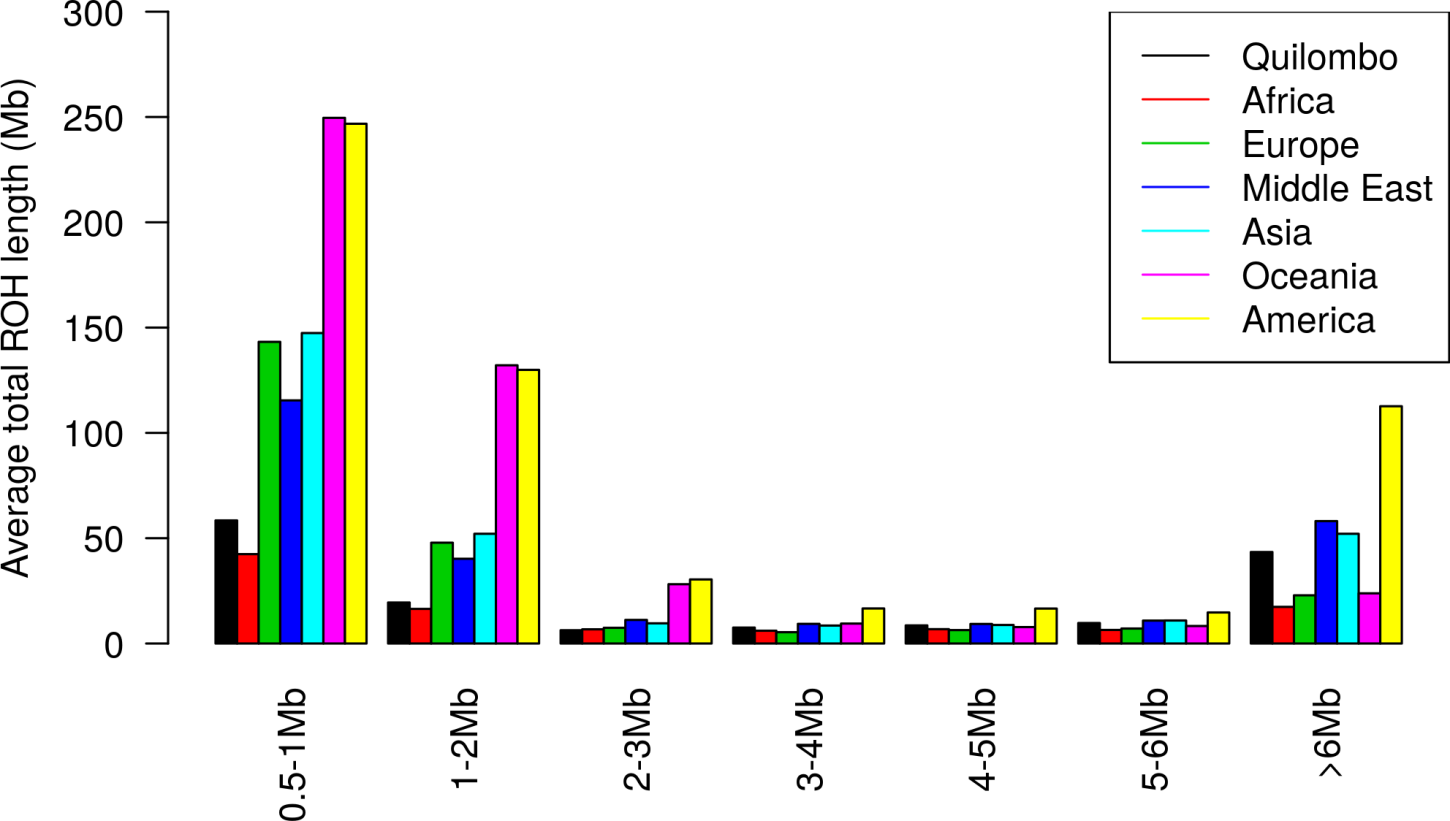
Total ROH length per individual. Distribution of individual average total ROH lengths according to continental regions.

When the individual patterns of ROH are analyzed (Fig 4), one notices that the average total ROH length from QUI composed by ROH lower than 1Mb is higher than African and smaller than European and American total lengths, which is expected since the isolate was founded by individuals of these three different ancestries and the amount of genomic ROH, especially those lower than 1Mb (which reflect the presence of ancient events that occurred in the parental populations), should be approximately proportional, but lower, to the genomic contribution of each parental population. This result suggests that LD patterns of admixed populations are strongly influenced by the LD patterns of the populations from which founder individuals originated.

Considering now the largest ROH (>6Mb), the QUI sample sums, in average, 50 Mb by individual, which is approximately twice the proportion observed for African and European samples. It highlights the occurrence of close inbreeding for at least part of the population and, less probably, the contribution of Native American ancestry components, that also harbor comparatively large portions of very long ROH. Since the foundation of the quilombo population is extremely recent (~8 generations), an interpretation for these results is that ROH <2Mb is probably still capturing non-recent inbreeding events that occurred in parental populations, and only the very large ROHs (certainly those >6Mb) reflect events occurring after origin of quilombo communities. Single ROH larger than 35Mb were found in seven (out of the total of 541) individuals.

**Fig 5:**
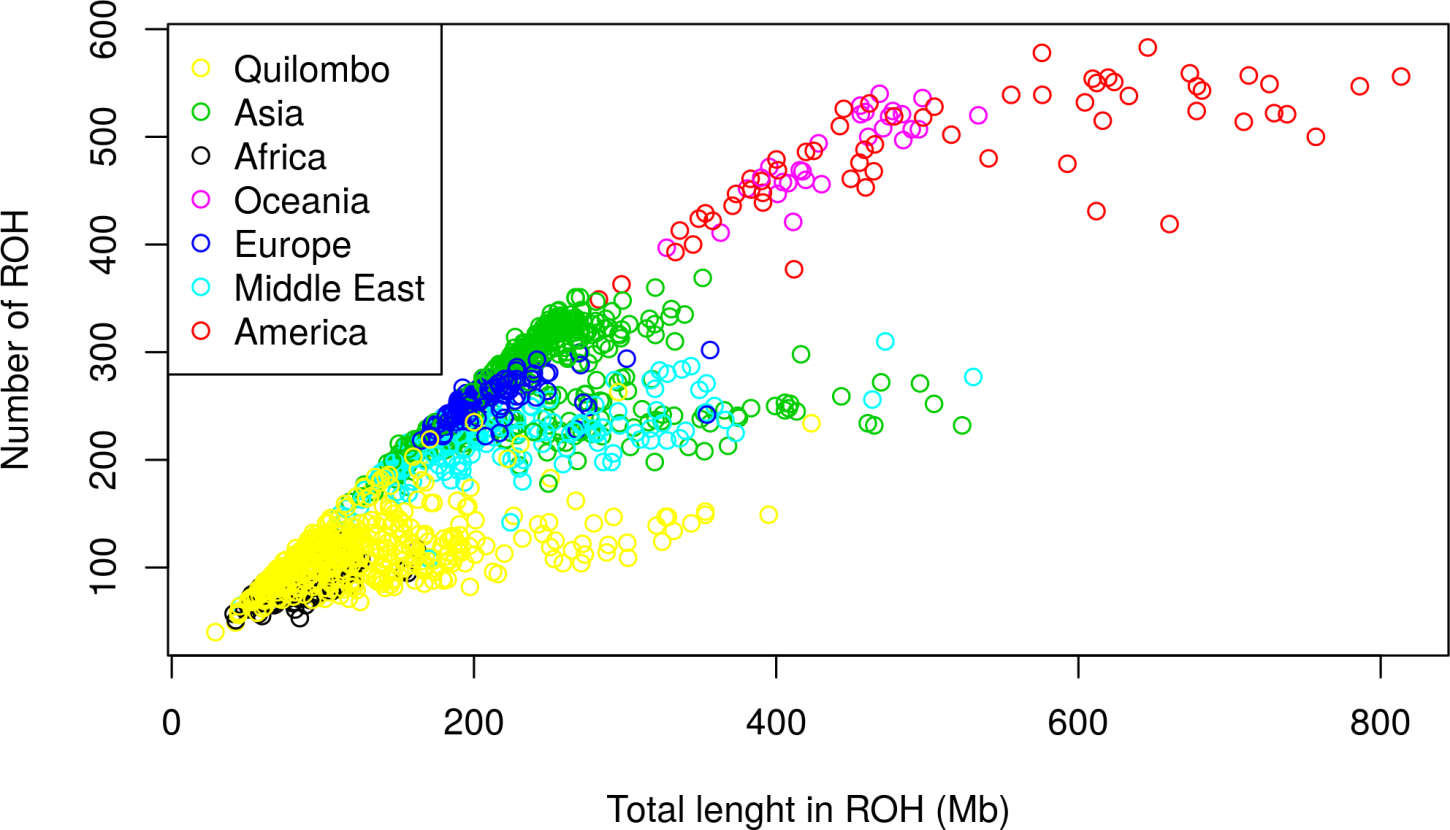
Individual patterns of ROH. The number of ROH compared to the total length in ROH for QUI and HGDP individuals according to continental regions and considering ROH above 0.5Mb.

We also plotted the numbers of ROH against their total lengths by continent (Fig 5), in order to obtain some additional information on demographic events occurring in the populations [18]. The QUI sample, as expected, showed to have, on average, a low number and a small total length of ROH, far lower than those observed for Native Americans, due probably to the occurrence of admixture, that inserts variability in the population. The presence of inbred QUI individuals is also suggested, since endogamous individuals have a proportionally high total ROH length, highlighted by a departure in the right direction from the main diagonal of the graph.

### Relationship between *f’* and *F*_*ROH*_ estimates

**Fig 6:**
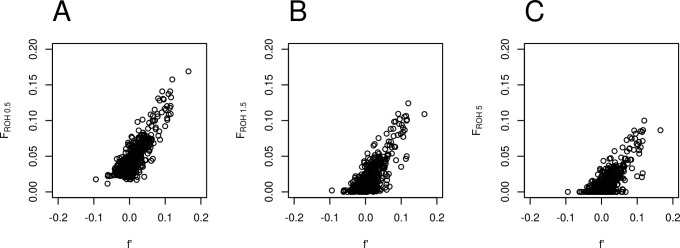
Comparison between individual inbreeding coefficient estimates. Scatter plots of individual estimates of inbreeding coefficient *f’* considering no-LD SNP dataset and *F*_*ROH*_ considering the sets of all ROH above (A) 0.5Mb, (B) 1.5Mb, and (C) 5 Mb.

The quilombo values of *f’* and *F*_*ROH*_ were estimated using two different techniques that should be correlated, since they are associated to the inbreeding levels of the population, corresponding to Wright's F_is_ and F_it_ respectively. The scatterplots of Fig 6 show the dispersion of individual values of corresponding pairs of *f’* (considering no-LD SNP dataset) and *F*_*ROH*_ considering the sets of all ROH above 0.5Mb (Pearson’s r = 0.8353, Spearman’s ρ = 0.7281), 1.5Mb (Pearson’s r = 0.7744, Spearman’s ρ = 0.6371), and 5Mb (Pearson’s r = 0.7554, Spearman’s ρ = 0.6102); all six correlation coefficients differ significantly from zero (P < 2.2×10^−16^).

The analysis above showed that *F*_*ROH 0*.*5*_ correlates better with *f’* than the other estimates, which makes sense, since smaller ROH are more informative on demographic events occurring before recent inbreeding.

## Discussion and conclusions

This study dealt with the issue of estimating parameters related to the system of marriages, inbreeding levels, and population/demographic events of a complex tri-hybrid admixed population.

Using information from both complete and no-LD datasets, we estimated Wright’s fixation index *F*_*IS*_ using two alternative methods. The first method obtains the population inbreeding coefficient averaging the fixation indices estimated from each locus of all sampled individuals; in the second one the population inbreeding coefficient is obtained by averaging the fixation indices indirectly obtained from all sampled loci of each individual. Our analyses showed that the lowest *var(F*_*IS*_*)* values might be pivotal in creating biased estimates of *F*_*IS*_-values even occurring in only a few markers; and that the optimal range of MAF for using in the estimation process in the QUI sample is in the range of 0.2 ≤ MAF ≤ 0.5. The two methods supplied reliable estimates with equivalent values, but since the second one can be directly applied without any further sample selection it is more convenient to use on practical grounds. Interestingly, the estimates we obtained do not diverge significantly from the ones obtained in a previous study of our group [1] using a far smaller number of markers (14 SNPs and 16 microsatellites) from the same population.

In relation to the ROH analysis, we used a reliable method to identify these regions in 52 populations (QUI plus 51 from HGDP), in order to occasionally obtain information about evolutionary forces acting in multiple time scales [7,30].

Taking into account ROH <2Mb, the quilombo population has an intermediate average total length of ROH when its parental population sources (Africa, Europe, and America) are considered. This suggests that the amount of shorter ROH is somehow proportional to the amount of corresponding ROH inherited from the parental stocks. Due to a complex admixture of individuals from different genomic sources, a factor that introduces genetic variability into the admixed population, its average fraction of shorter ROH should be lower than (but still proportional to) the real contributions from each parental stock.

For homozygous segments larger than 6Mb, the total average lengths of ROH obtained from QUI showed to be approximately twice the estimates from Africa and Europe, reflecting the presence of a very recent and significant amount inbreeding.

We also detected significant positive correlation coefficients between the individual estimates of *F*_*ROH*_ and *F*_*IS*_, especially when the set of all ROH above 0.5Mb was considered.

## Supporting information

S1 Fig Estimates of per locus inbreeding coefficient values. (A) complete dataset; (B) no-LD dataset.
(TIFF)

S2 Fig Distribution of the variance of inbreeding coefficient according to MAF. Distribution of per locus *var(f*_*k*_*)* estimates according to MAF intervals for the complete dataset. (A) 0–0.1; (B) 0.1–0.2; (C) 0.2-0.3; (D) 0.3–0.4; (E) 0.4–0.5.
(TIFF)

S1 Table Numbers of genotyped individuals at a given community. Communities are as defined in Fig 1; N, estimated number of adult individuals [31]; N_G_, number of genotyped individuals; n_G_, percentage of genotyped individuals.
(DOCX)

S2 Table Estimates of inbreeding coefficient from ROH by population. Mean, median and corresponding observed 95% confidence intervals of individual inbreeding coefficients *F*_*ROH*_ per continent, considering ROH above 1.5Mb. The estimates were made considering 52 populations (QUI plus 51 from HGDP).
(DOCX)
