# Supplementary figures and images for: Inbreeding estimates in human populations: Applying new approaches to an admixed Brazilian isolate
Source: PLoS One. 2018 Apr 24;13(4):e0196360. doi: 10.1371/journal.pone.0196360 (PMC5916862; doi:10.1371/journal.pone.0196360)

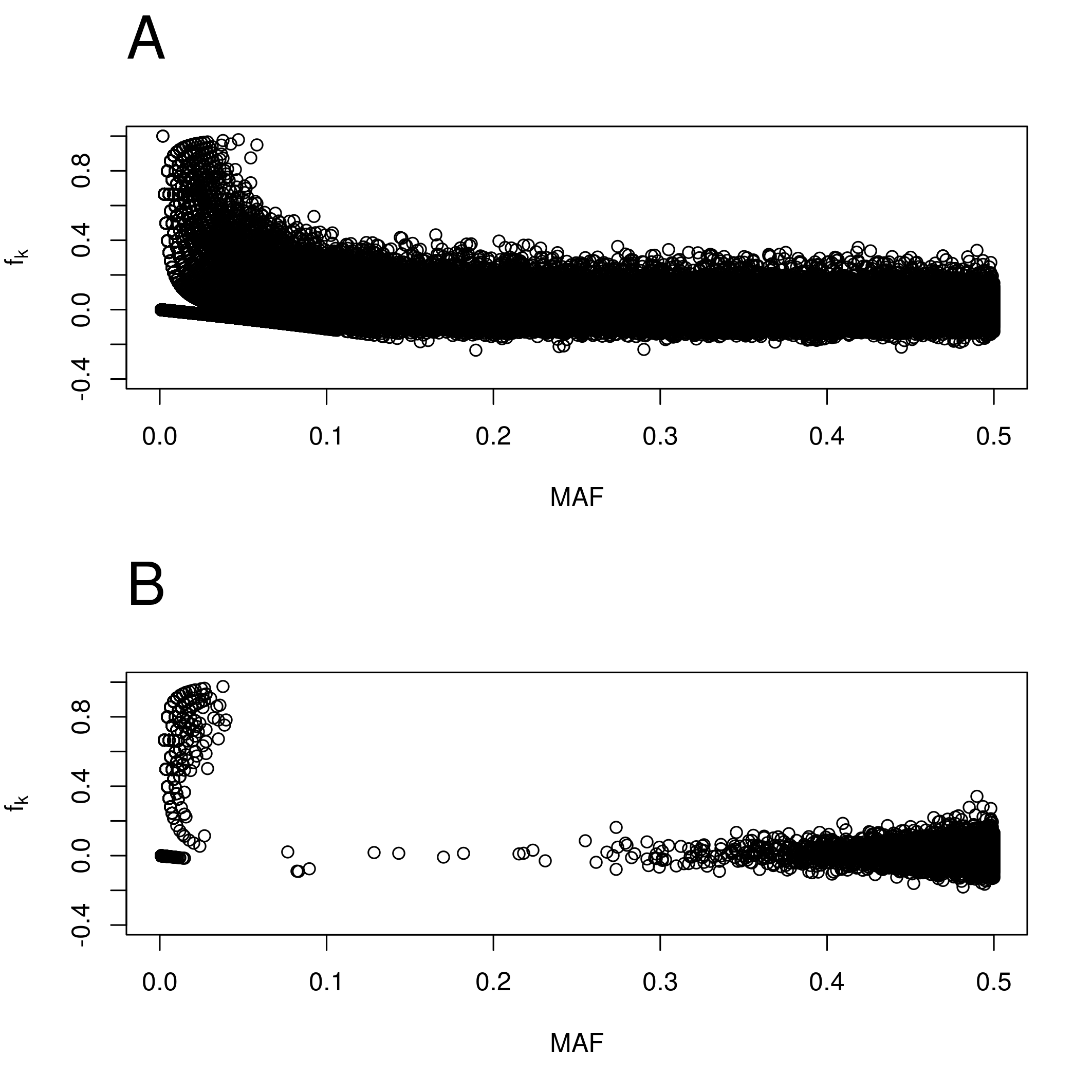

Supplement: S1 Fig — (A) complete dataset; (B) no-LD dataset. (TIFF) [file pone.0196360.s001.tiff]

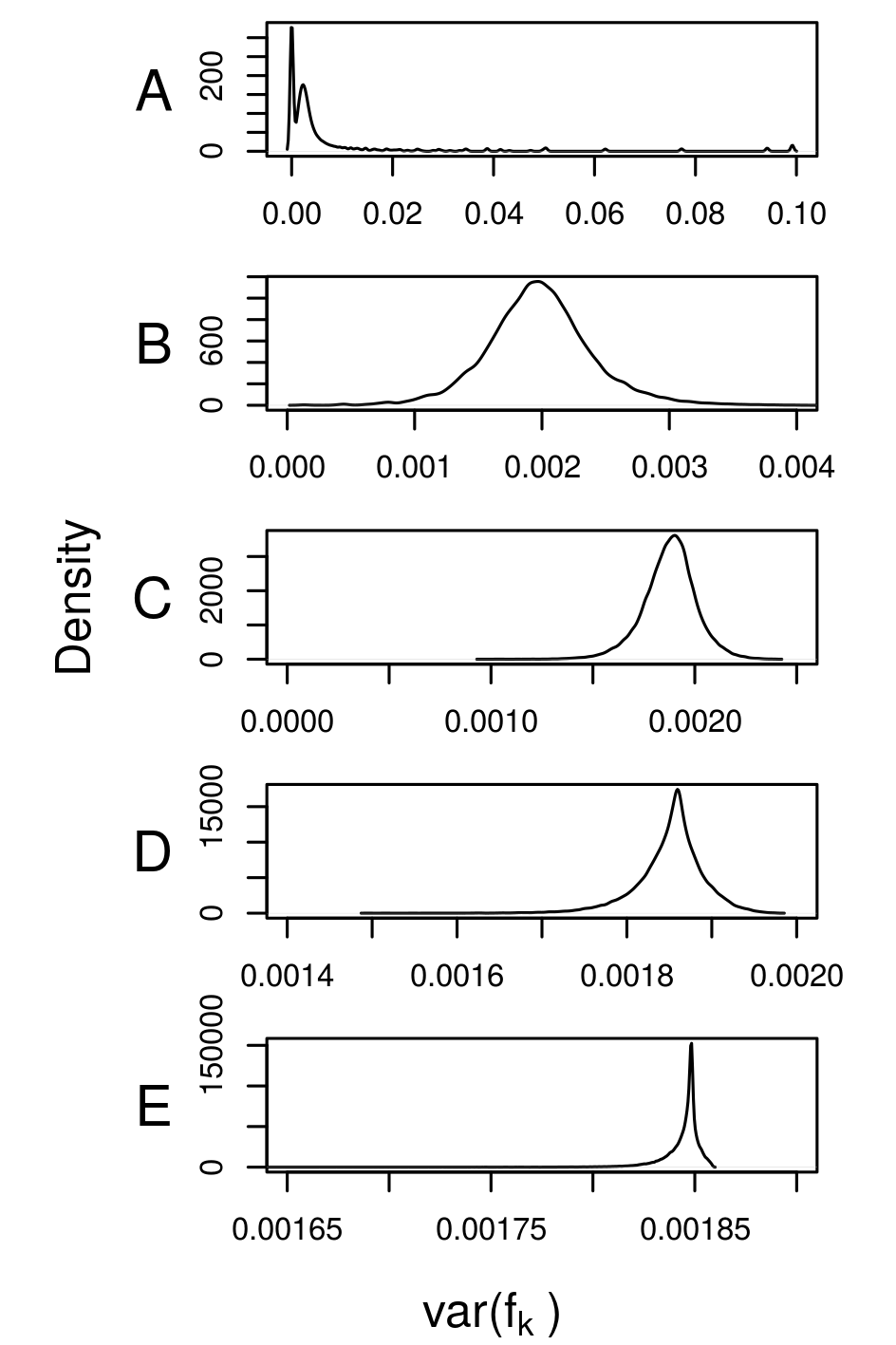

Supplement: S2 Fig — Distribution of per locus var(fk) estimates according to MAF intervals for the complete dataset. (A) 0–0.1; (B) 0.1–0.2; (C) 0.2-0.3; (D) 0.3–0.4; (E) 0.4–0.5. (TIFF) [file pone.0196360.s002.tiff]
