# Supplementary material for: Inbreeding estimates in human populations: Applying new approaches to an admixed Brazilian isolate
Source: PLoS One. 2018 Apr 24;13(4):e0196360. doi: 10.1371/journal.pone.0196360 (PMC5916862; doi:10.1371/journal.pone.0196360)
Supplement: S1 Table — Communities are as defined in Fig 1; N, estimated number of adult individuals [31]; NG, number of genotyped individuals; nG, percentage of genotyped individuals. (DOCX) [file pone.0196360.s003.docx]

S1 Table. Numbers of Genotyped Individuals at a Given Community.

|  | *AB* | *AN* | *GA* | *IV* | *MR* | *NH* | *PA* | *PC* | *PS* | *RE* | *SP* | *TU* | Total |
| --- | --- | --- | --- | --- | --- | --- | --- | --- | --- | --- | --- | --- | --- |
| *N* | 573 | 320 | 134 | 270 | 56 | 447 | 220 | 286 | 128 | 250 | 132 | 295 | 3111 |
| *N_G_* | 95 | 75 | 37 | 44 | 10 | 39 | 26 | 55 | 34 | 28 | 43 | 55 | 541 |
| *n_G_* | 16.6 | 23.4 | 27.6 | 16.3 | 17.9 | 8.7 | 11.8 | 19.2 | 26.6 | 11.2 | 32.6 | 18.6 | 17.4 |

Communities are as defined in Figure 1; *N*, estimated number of adult individuals [3]; *N*_G_, number of genotyped individuals; *n_G_*, percentage of genotyped individuals.
