# Supplementary material for: Inbreeding estimates in human populations: Applying new approaches to an admixed Brazilian isolate
Source: PLoS One. 2018 Apr 24;13(4):e0196360. doi: 10.1371/journal.pone.0196360 (PMC5916862; doi:10.1371/journal.pone.0196360)
Supplement: S2 Table — Mean, median and corresponding observed 95% confidence intervals of individual inbreeding coefficients FROH per continent, considering ROH above 1.5Mb. The estimates were made considering 52 populations (QUI plus 51 from HGDP). (DOCX) [file pone.0196360.s004.docx]

S2 Table. Estimates of inbreeding coefficient from ROH by population.

| *Region* | *Population* | *Mean* | *Median* | *var(F)* | *Observed 95% c.i.* |
| --- | --- | --- | --- | --- | --- |
| Quilombo | Quilombo | 0.0193 | 0.0111 | 0.000495 | (0.0007, 0.0882) |
| Africa | Bantu | 0.0070 | 0.0051 | 0.000024 | (0.0023, 0.0163) |
| Africa | Biaka_Pygmies | 0.0159 | 0.0158 | 0.000058 | (0.0028, 0.0236) |
| Africa | Mandenka | 0.0048 | 0.0021 | 0.000063 | (0.0007, 0.0119) |
| Africa | Mbuti_Pygmies | 0.0144 | 0.0120 | 0.000066 | (0.0058, 0.0235) |
| Africa | San | 0.0184 | 0.0192 | 0.000070 | (0.0079, 0.0252) |
| Africa | Yoruba | 0.0036 | 0.0023 | 0.000020 | (0.0006, 0.0078) |
| America | Colombians | 0.0436 | 0.0353 | 0.000938 | (0.0196, 0.0403) |
| America | Karitiana | 0.1067 | 0.1017 | 0.000892 | (0.0676, 0.1376) |
| America | Maya | 0.0240 | 0.0215 | 0.000125 | (0.0111, 0.0449) |
| America | Pima | 0.0727 | 0.0542 | 0.001450 | (0.0264, 0.1280) |
| America | Surui | 0.1471 | 0.1488 | 0.000832 | (0.0914, 0.1786) |
| Asia | Balochi | 0.0497 | 0.0448 | 0.001820 | (0.0033, 0.1244) |
| Asia | Brahui | 0.0329 | 0.0179 | 0.001259 | (0.0049, 0.1051) |
| Asia | Burusho | 0.0141 | 0.0121 | 0.000118 | (0.0026, 0.0384) |
| Asia | Cambodians | 0.0077 | 0.0080 | 0.000003 | (0.0050, 0.0095) |
| Asia | Dai | 0.0125 | 0.0125 | 0.000038 | (0.0051, 0.0224) |
| Asia | Daur | 0.0113 | 0.0112 | 0.000014 | (0.0069, 0.0141) |
| Asia | Han | 0.0083 | 0.0082 | 0.000006 | (0.0051, 0.0120) |
| Asia | Hazara | 0.0157 | 0.0110 | 0.000174 | (0.0038, 0.0390) |
| Asia | Hezhen | 0.0128 | 0.0090 | 0.000123 | (0.0036, 0.0198) |
| Asia | Japanese | 0.0117 | 0.0103 | 0.000042 | (0.0043, 0.0251) |
| Asia | Kalash | 0.0373 | 0.0380 | 0.000217 | (0.0069, 0.0563) |
| Asia | Lahu | 0.0283 | 0.0274 | 0.000268 | (0.0092, 0.0425) |
| Asia | Makrani | 0.0437 | 0.0372 | 0.001700 | (0.0030, 0.1128) |
| Asia | Miaozu | 0.0158 | 0.0147 | 0.000028 | (0.0093, 0.0218) |
| Asia | Mongola | 0.0070 | 0.0067 | 0.000005 | (0.0040, 0.0096) |
| Asia | Naxi | 0.0130 | 0.0134 | 0.000016 | (0.0074, 0.0180) |
| Asia | Oroqen | 0.0137 | 0.0113 | 0.000062 | (0.0041, 0.0236) |
| Asia | Pathan | 0.0318 | 0.0070 | 0.001866 | (0.0013, 0.1348) |
| Asia | She | 0.0171 | 0.0166 | 0.000032 | (0.0082, 0.0236) |
| Asia | Sindhi | 0.0299 | 0.0119 | 0.000940 | (0.0021, 0.0799) |
| Asia | Tu | 0.0073 | 0.0071 | 0.000005 | (0.0041, 0.0100) |
| Asia | Tujia | 0.0183 | 0.0080 | 0.000677 | (0.0045, 0.0323) |
| Asia | Uygur | 0.0069 | 0.0042 | 0.000093 | (0.0009, 0.0066) |
| Asia | Xibo | 0.0068 | 0.0076 | 0.000005 | (0.0029, 0.0086) |
| Asia | Yakut | 0.0248 | 0.0253 | 0.000086 | (0.0090, 0.0380) |
| Asia | Yizu | 0.0122 | 0.0098 | 0.000093 | (0.0026, 0.0212) |
| Europe | Adygei | 0.0102 | 0.0078 | 0.000081 | (0.0028, 0.0225) |
| Europe | French | 0.0082 | 0.0054 | 0.000123 | (0.0015, 0.0444) |
| Europe | French_Basque | 0.0153 | 0.0135 | 0.000118 | (0.0060, 0.0228) |
| Europe | North_Italian | 0.0131 | 0.0067 | 0.000431 | (0.0043, 0.0211) |
| Europe | Orcadian | 0.0106 | 0.0087 | 0.000026 | (0.0037, 0.0167) |
| Europe | Russian | 0.0082 | 0.0070 | 0.000017 | (0.0031, 0.0162) |
| Europe | Sardinian | 0.0147 | 0.0114 | 0.000066 | (0.0052, 0.0285) |
| Europe | Tuscan | 0.0077 | 0.0050 | 0.000041 | (0.0021, 0.0070) |
| Middle East | Bedouin | 0.0400 | 0.0374 | 0.000782 | (0.0010, 0.0793) |
| Middle East | Druze | 0.0331 | 0.0220 | 0.000960 | (0.0036, 0.0802) |
| Middle East | Mozabite | 0.0228 | 0.0192 | 0.000217 | (0.0023, 0.0540) |
| Middle East | Palestinian | 0.0202 | 0.0095 | 0.000553 | (0.0025, 0.0559) |
| Oceania | NAN_Melanesian | 0.0322 | 0.0317 | 0.000083 | (0.0195, 0.0430) |
| Oceania | Papuan | 0.0415 | 0.0415 | 0.000152 | (0.0184, 0.0559) |

Mean, median and corresponding observed 95% confidence intervals of individual inbreeding coefficients *F_ROH_* per continent, considering ROH above 1.5Mb. The estimates were made considering 52 populations (QUI plus 51 from HGDP).
